# Marine Microorganism Molecules as Potential Anti-Inflammatory Therapeutics

**DOI:** 10.3390/md22090405

**Published:** 2024-09-03

**Authors:** Malia Lasalo, Thierry Jauffrais, Philippe Georgel, Mariko Matsui

**Affiliations:** 1 Group Bioactivities of Natural Compounds and Derivatives (BIONA), Institut Pasteur of New Caledonia, Member of the Pasteur Network, Noumea 98845, New Caledonia; mlasalo@pasteur.nc; 2 Ifremer, Institut de Recherche pour le Développement (IRD), Centre Nationale de la Recherche Scientifique (CNRS), Université de la Réunion, Université de la Nouvelle-Calédonie, UMR 9220 ENTROPIE, 101 Promenade Roger Laroque, Noumea 98897, New Caledonia; thierry.jauffrais@ifremer.fr; 3 Team Neuroimmunology and Peptide Therapy, Biotechnologie et Signalisation Cellulaire, UMR 7242, University of Strasbourg, 67085 Strasbourg, France; pgeorgel@unistra.fr

**Keywords:** anti-inflammatory, inflammation, microorganisms, MNPs

## Abstract

The marine environment represents a formidable source of biodiversity, is still largely unexplored, and has high pharmacological potential. Indeed, several bioactive marine natural products (MNPs), including immunomodulators, have been identified in the past decades. Here, we review how this reservoir of bioactive molecules could be mobilized to develop novel anti-inflammatory compounds specially produced by or derived from marine microorganisms. After a detailed description of the MNPs exerting immunomodulatory potential and their biological target, we will briefly discuss the challenges associated with discovering anti-inflammatory compounds from marine microorganisms.

## 1. Introduction

Chronic inflammatory diseases (CIDs) have emerged as a significant global concern, with a prevalence of 5 to 7% of Western society in 2010 [1]. These illnesses, such as psoriasis, rheumatoid arthritis (RA), inflammatory bowel disease (IBD), Crohn’s disease (CD), or ulcerative colitis (UC), can be debilitating, leading to a reduced quality of life and, in the most severe cases, premature death [2].

Conventional treatments based on corticoids and non-steroidal anti-inflammatory drugs (NSAIDs) often lead to severe side effects, including gastrointestinal ulceration and bleeding, osteoporosis, hypertension, and glaucoma. Drug development more recently has focused on monoclonal antibodies targeting inflammatory cytokines such as tumor necrosis factor-α (TNF-α) or interleukins (e.g., IL-6) [3], or inhibitors of pathways activated by inflammatory cytokines, such as Janus Kinase inhibitors (Jakinibs) [4]. Although these therapies have shown considerable clinical efficacy, many patients remain unresponsive, and others may develop resistance to monoclonal antibody treatment. Furthermore, the use of such immunomodulatory molecules carries a limited but notable risk of developing opportunistic infections, such as Herpes Zoster Virus [5].

As life expectancy increases, there is an increased likelihood of developing CIDs, and therefore, managing these diseases has become more challenging. Hence, continuing to explore innovative treatment exploration and improving their response to these debilitating diseases is crucial. In this regard, the discovery of bioactive molecules from marine microorganisms represents a groundbreaking pharmaceutical development that could promote the identification of novel therapeutic compounds to treat CIDs.

Here, we aim to review marine microorganisms that produce molecules with potential pharmaceutical relevance, categorizing them based on producing genus and species, compounds’ molecular structures, and their mechanism of action on immune signaling pathways. Additionally, we will provide a brief overview of the difficulties related to identifying anti-inflammatory compounds derived from marine microorganisms.

While previous reviews have primarily centered on symbiotic bacteria, to the best of our knowledge, none have yet highlighted the anti-inflammatory properties of these microorganisms. For this review, we selected 208 articles published from 2000 to 2024. One anterior reference was retained for the historical aspect of a specific molecule. The search engines Google Scholar, Science Direct, PubMed, and MarinLit databases were used with the keywords “marine natural products” combined with “anti-inflammatory”, “macro-organisms”, “microorganisms”, “clinical pipeline”, “clinical use”, and “bioactivities.” The database Worms (https://www.marinespecies.org/, accessed on 17 January 2024) was used to identify the species of marine organisms.

## 2. The Link between the Inflammation and CIDs

Harmful stimuli such as pathogens, toxic compounds, injuries, or irradiation induce cell damage and trigger an inflammatory response, a crucial component of our innate immune system [6]. This process involves the detection of danger signals that are recognized by dedicated immune receptors [7], enabling the elimination of such unwanted signals and the initiation of the healing process, thereby maintaining tissue homeostasis and a healthy condition. However, this process requires strict control and must be initiated locally and temporarily. In fact, systemic and chronic inflammations are associated with most human diseases and mortality [2]. Although some features of inflammatory responses may vary depending on the initial stimulus and its location in the body, they are characterized by dedicated signaling pathways and transcriptional signatures.

### 2.1. Inflammatory Pathways

Deciphering the regulatory pathways and mediators involved in inflammation is crucial for developing effective treatments against various diseases. A central player in inflammation is the NF-κB transcription factor, which controls the production of pro-inflammatory cytokines and, subsequently, the recruitment of immune cells. The nuclear translocation of NF-κB is regulated by IκB, which, once phosphorylated by upstream kinases in response to innate immune receptor engagement, is degraded by the proteasome (reviewed in [8]). In the case of IBD, the overactivation of this pathway directly causes an increase in the production of pro-inflammatory cytokines such as TNF-α, IL-1, and IL-6, consequently fueling chronic inflammation [9].

Similarly, Mitogen-activated Protein Kinases (MAPKs) are a family of protein kinases that respond to various stimuli, including inflammatory cytokines. They influence cell proliferation, differentiation, survival, and apoptosis. The activation of MAPKs leads to the phosphorylation and activation of p38 transcription factors, which also activate inflammatory response genes [10]. In the joint tissue of RA patients, the mentioned pathway regulates the production of pro-inflammatory cytokines. Also, it has a crucial role in the signaling cascade downstream of interleukin (IL-1), IL-17, and TNF-α, leading to cartilage destruction [11].

The JAK-STAT pathway is another highly conserved signaling mechanism significantly regulating inflammatory gene expression. Upon ligands (which are primarily cytokines, such as interferons) binding to their cognate receptors, intracellular receptor-associated Janus-activated kinases (JAKs) phosphorylate each other and dimerize, creating docking sites for Signal Transducers and Activators of Transcription (STATs), which are latent, cytoplasmic transcription factors. The cytoplasmic STATs undergo phosphorylation and subsequent dimerization, enabling their translocation to the nucleus, where they modulate immune-related gene expression [12]. Under normal conditions, this pathway is governed by negative regulators of JAK/STAT, including the suppressor of cytokine signaling and protein inhibitor of activated STAT. However, in the context of rheumatoid arthritis (RA), the malfunction of these regulators leads to joint damage commonly observed in affected patients [13].

Finally, inflammasome (among which is the NOD-like receptor family, the pyrin domain containing three signaling, or NLRP3 is the best described) signaling is also activated during many inflammatory responses. Inflammasomes require a sensor, an adaptor, and a pro-caspase that, following puncta formation, leads to IL-1β secretion, an important player in several (auto) inflammatory disorders, such as gouty arthritis [14].

Because dysregulation of NF-κB, MAPKs, JAK-STAT, or inflammasomes activity is often associated with inflammatory, autoimmune, or metabolic diseases, a thorough investigation of the corresponding pathways offers tremendous opportunities to develop more effective treatments for these diseases and improve patient outcomes.

### 2.2. Therapeutic Strategies to Target Inflammation

Until the end of the 20th century, CIDs therapeutics relied essentially on glucocorticoids and other small chemicals (non-steroidal) based on their anti-inflammatory, immunomodulatory, or anti-proliferative properties. Over the past 20 years, the management of patients who have rheumatoid arthritis (RA), one of the most frequent CIDs, witnessed significant improvements with the development and marketing of biologic and targeted-synthetic disease-modifying antirheumatic drugs (b/tsDMARDs). These molecules are designed to target and neutralize cytokines (such as TNF-α) and their receptors, to deplete specific cell populations (such as B lymphocytes with the anti-CD20 antibody), to modulate T cells activation (using the CTLA4-Ig) or to impact signaling pathways (with JAK inhibitors for instance) [15].

In this regard, TNF-α inhibitors completely changed the therapeutic strategy of RA patients, moving from relieving their symptoms to complete remission, which is the goal of the current therapy.

However, despite that considerable progress, many unmet clinical needs persist for CID patients. Indeed, even in the case of RA, a significant proportion of patients remain refractory to available therapies, and others develop resistance to effective drugs (as can be observed following anti-TNF-α treatment) [16]. For IBD patients, ~10% to 30% of patients resist the anti-TNF-α agent (primary non-responder), and 20% to 50% of responding patients (secondary loss of response) develop a resistance to the treatment within one year [17]. In addition, many chronic inflammatory syndromes (like scleroderma or Sjögren syndrome) are still without any reference treatment [18]. Therefore, the search for alternative therapeutic options remains current.

**Table 1:** MNPs with anti-inflammatory activity. ?: no species identified.

Macro-Organisms						
Organisms	Classification (Phylum)	Species	Type of Molecules	Molecules	Target/Mode of Action	Ref(s).
Sponge	Porifera	*Fasciospongia cavernosa*	Terpene lactone	Cavernolide	TNF-α, NO, and PGE2 inhibition in RAW 264.7 cells	[19]
Sponge	Porifera	*Dysidea* spp.	Sesquiterpene	Dysidotronic acid	TNF-α, IL-1, NO, PGE2 inhibition in RAW 264.7 cells	[20]
Sponge	Porifera	*Plakortis* spp.	α-exomethylene-γ-lactone	Plakolide A	iNOS inhibition in RAW 264.7 cells	[21]
Sponge	Porifera	*Luffariella variabilis*	Sesterterpene	Manoalide	Eicosanoids synthesis inhibition in human polymorphonuclear leukocytes	[22]
Caribbean sponge	Porifera	*Cacospongia linteiformis*	Sesterterpene	Cyclolinteinone	iNOS and COX-2 inhibition in LPS-stimulated J774 macrophages	[23]
Sponge	Porifera	*Dysidea* sp. and *Petrosaspongia nigra*	Merosesquiterpene & Sesterterpene	Bolinaquinone and petrosaspongiolide M	Protection against TNBS-induced colitis in BALB/c mice	[24]
Sponge	Porifera	*Petrosia* spp.	Polyacetylenes	Petrocortyne D, Petrocortyne E, Petrocortyne F, Petrocortyne G, Petrocortyne H	Inhibition of PLA2 activity in K-562 cell line	[25]
Sponge	Porifera	*Petrosia* spp.	Polyacetylenic alcohol	Petrocortyne A	TNF-α inhibition in LPS-activated RAW 264.7 and PMA/LPS-treated U937 cells and NO inhibition in LPS- or IFNγ-treated RAW 264.7 cells	[26]
Sponge	Porifera	*Theonella swinhoe*	Steroid	Solomonsterol A	Reduction in arthritic score in anti-type II collagen antibody-induced arthritis murine model	[27]
Sponge	Porifera	*Geodia barretti*	Alpha amino acids and derivatives	Barettin	TNF-α and IL-1β inhibition in LPS-stimulated THP-1 cells	[28]
Sponge	Porifera	*Geodia barretti*	Alkaloids	6-bromoindole derivatives geobarettin B, 6-bromoindole derivatives geobarettin C, 6-bromoindole alkaloids 6-bromoconicamin, barettin	IL-12 p40 inhibition and IL-10 increasing in dendritic cells	[29]
Sponge	Porifera	*Halichondria okadai*	Alkaloid	Halichlorine	VCAM-1, ICAM-1, and E-selectin inhibition in LPS-stimulated aortic endothelial cells, inhibition of macrophage adhesion to cultured cell monolayers, an anti-inflammatory effect associated with NF-κB pathway	[30]
Sponge	Porifera	*Stylissa*	Alkaloid	Pyrrole alkaloid (10Z)-debromohymenialdisine	IL-1β, IL-6, TNF-α, iNOS, COX-2, NO and PGE2 inhibition in co-cultures of LPS-stimulated Caco-2 and THP-1 cells	[31]
Sponge	Porifera	*Stylissa flabellata*	Alkaloids	Stylissadine A, Stylissadine B	Antagonistic effect on P2X7 receptors in THP-1 cells	[32]
Soft coral	Cnidaria	*Sinularia dissecta*	Diterpene	Seco-sethukarailin	Inhibition of pro-inflammatory cytokines in bone marrow-derived dendritic cells	[33]
Soft coral	Cnidaria	*Pseudopterogorgia elisabethae*	Diterpenes	Pseudopterosin E, Pseudopterosin A	Reduction of PMA-induced mouse ear edema; PGE2 and LCT4 inhibition in zymosan-stimulated murine peritoneal macrophages	[34]
Soft coral	Cnidaria	*Sinularia gibberosa*	Steroid	Gibberoketosterol	Inhibition of pro-inflammatory iNOS and COX-2 proteins in LPS-stimulated RAW264.7 cells	[35]
Okinawan soft coral	Cnidaria	*Sinularia* spp.	Diterpenes	Norcembranolide and sinuleptolide	TNF-α and NO inhibition in LPS-stimulated RAW 264.7 cells	[36]
Soft coral	Cnidaria	*Sinularia lochmodes*	Sesquiterpene	Lochmolins A, Lochmolins B	Inhibition of COX-2 expression in LPS-activated RAW 264.7 cells	[37]
				Lochmolins C	Inhibition of COX-2 expression in LPS-activated RAW 264.7 cells	[38]
				Lochmolins D	Inhibition of COX-2 expression in LPS-activated RAW 264.7 cells	[37]
Soft coral	Cnidaria	*Lemnalia cervicorni*	Sesquiterpene	Lemnalol	Inhibition of iNOS and COX-2 expression in LPS-activated RAW 264.7 cells; inhibition of iNOS and COX-2 expression in carrageenan-activated rat paws	[39]
Soft coral	Cnidaria	*Lemnalia flava*	Sesquiterpene	Flavalin A	iNOS and COX-2 inhibition in RAW 264.7 cells	[40]
Soft coral	Cnidaria	*Lobophytum crassum*	Diterpenes	Crassumol E 1R,4R,2E,7E,11E-cembra-2,7,11-trien-4-ol	Inhibition of NF-κB activation in TNF-α-activated HepG2 cells	[41]
			Diterpenes	Lobocrasol A, Lobocrasol B	Inhibition of NF-κB activation in TNF-α-activated HepG2 cells	[42]
Soft coral	Cnidaria	*Scleronephthya gracillimum*	Steroid	Sclerosteroid J	Inhibition of iNOS and COX-2 expression in LPS-activated RAW 264.7 cells	[43]
Octocoral	Cnidaria	*Pseudopterogorgia acerosa*	Diterpene	Pseudopterane	Inhibition of NO, TNF-α, IL-1β and IFNγ-induced protein production in LPS-activated peritoneal macrophages	[44]
Coral	Cnidaria	*Rumphella antipathies* (classification *rhumphella antipathes* Linnaeus 1758)	Sesquiterpene	Clovane compound 1	Inhibition of superoxide anions generation and elastase release	[45]
			Sesquiterpene	Clovane compound 2	Inhibition of elastase release in fMLP/CB-activated human neutrophils	[45]
			Sesquiterpene	Rumphellaone C	Inhibition of superoxide anion generation and elastase release in human neutrophils	[46]
			Sesquiterpene	Rumphellol A	Inhibition of superoxide generation and elastase release in human neutrophils	[47]
			Sesquiterpene	Rumpheloll B		
Coral	Cnidaria	*Briareum excavatum*	Diterpene	Excavatolide B	Inhibition of iNOS expression in carrageenan-activated rat paws	[48]
Coral	Cnidaria	*Briareum excavatum*	Diterpene	Excavatolide B	Inhibition of 12-O-tetradecanoylphorbol-13-acetate (TPA)-induced vascular permeability; inhibition of TPA-induced matrix metalloproteinase-9 expression in mouse skin; inhibition of IL-6 expression of LPS-activated mouse bone marrow-derived dendritic cells	[49]
Anemone	Cnidaria	*Zoanthus kuroshio*	Alkaloid	5α-iodozoanthenamine	Anti-inflammatory effect on—neutrophils, reduction of superoxide anion generation, and elastase by cells	[50]
Anemone	Cnidaria	*Zoanthus pulchellus*	Alkaloids	3-hydroxinorzoanthamine Norzoanthine Roanthamine	ROS and NO inhibition in LPS-stimulated BV-2 cells	[51]
Starfish	Echinodermata	*Marthasterias glacialis*	Steroid	Ergosta-7,22-dien-3-ol	Inhibition of iNOS protein level in LPS-activated RAW 264.7 cells	[52]
Starfish	Echinodermata	*Astropecten polycanthus*	Steroid	Steroid compound 5	Inhibition of IL-12 p40, IL-6, and TNF-α production in LPS-activated mice bone marrow-derived dendritic cells	[53]
Starfish	Echinodermata	*Asterias amurensis*	Fatty acid	Fatty acids	Inhibition of the expression of inflammatory genes via NF-κB and MAPK pathways in LPS-stimulated RAW 264.7 cells	[54]
Starfish	Echinodermata	*Marthasterias glacialis*	Fatty acid	Cis 11-eicosenoic and cis 11,14 eicosadienoic acids	Inhibition of iNOS, COX-2, IκBα, and NF-κB gene expression in LPS-stimulated RAW 264.7 cells	[52]
Starfish	Echinodermata	*Protoreaster nodosus*	Steroid	Oxygenated steroid derivatives	IL-12 p40, IL-6, and TNF-α inhibition in bone marrow-derived dendritic cells	[55]
Starfish	Echinodermata	*Protoreaster lincki*	Steroids	Protolinckioside A, Protolinckioside B, Protolinckioside C, Protolinckioside D	Reduction of ROS formation and NO production in LPS-stimulated RAW 264.7 cells	[56]
Starfish	Echinodermata	*Anthenea aspera*	Steroid	Anthenoside O		[57]
Starfish	Echinodermata	*Pentaceraster regulus*	Steroid	Pentareguloside C, Pentareguloside D, Pentareguloside E	Reduction of ROS formation and NO production in LPS-stimulated RAW 264.7 cells	[58]
Starfish	Echinodermata	*Acanthaster planci*	Pyrrole oligoglycoside	Plancipyrroside A, Plancipyrroside B	Reduction of ROS formation and NO production in LPS-stimulated RAW 264.7 cells	[59]
Starfish	Echinodermata	*Asterina batheri*	Pyrrole oligoglycoside	Astebatherioside B, Astebatherioside C, Astebatherioside D	IL-12 p40 inhibition in LPS-stimulated bone marrow-derived dendritic cells	[60]
Sea cucumber	Echinodermata	*Holothuria grisea*	Protein	Lectin	Inhibition of neutrophil migration to the peritoneal cavity in carrageenan-activated rats; reduction of myeloperoxidase activity in carrageenan-activated rats	[61]
Sea cucumber	Echinodermata	*Apostichopus japonicus* and *Stichopus chloronotus*	Sulfated polysaccharide	Fucosylated chondroitin sulfate	Reduction of neutrophil migration, inhibition of paw edema in carrageenan-induced paw edema in rats	[62]
Sea cucumber	Echinodermata	*Isostichopus badionotus*	Sulfated polysaccharide	Fucosylated chondroitin sulfate	Suppression of TPA-mediated up-regulation of TNF-α, IL-6, NF-κB, iNOS, IL-10, IL-11, COX-2 and STAT3 genes in mouse ear tissue	[63]
Sea cucumber	Echinodermata	*Isostichopus badionotus*	Sulfated polysaccharide	Fucoidan	Regulation of serum inflammatory cytokines (TNF-α, CRP, MIP-1, IL-1β, IL-6, and IL-10) and their mRNA expression, inactivation of JNK and IκB/NF-κB pathways	[64]
Sea cucumber	Echinodermata	*Holothuria albiventer* and *Cucumaria frondosa*	Sulfated polysaccharide	Sulfated fucan/FCS	Suppression of TNF-α and IL-6 production	[65]
Sea cucumber	Echinodermata	*Holothuria tomasi*	Triterpenes glycoside		Inhibition of IL-6, TNF-α levels in STZ-induced diabetic rats	[66]
Sea cucumber	Echinodermata	*Pearsonothuria graeffei*	Triterpenes glycoside	Holothurin A and Echinoside A	Inhibition of IL-1β, TNF-α, IL-6 and infiltration of macrophages in obese mice via p-ERK/cPLA2/COX-1 pathway and reduction of the PGE2 levels	[67]
Sea cucumber	Echinodermata	*Aspostichopus japonicus* and *Acaudina leucoprocta*	Peptide	Oligopeptides	Downregulation of pro-inflammatory cytokines transcription, upregulation of anti-inflammatory cytokines, and inhibition of TLR4/MyD88/NF-κB signaling pathway	[68]
Sea cucumber	Echinodermata	*Cucumaria frondosa*	Fatty acid	Eicosapentaenoic acid	Inhibition of TNF-α, IL-6, and MCP1 expression, attenuation of macrophage infiltration in the liver in mice, attenuation of the phosphorylation of NF-κB in RAW 264.7 cells	[69]
Sea cucumber	Echinodermata	*Cucumaria frondosa*	Lipid	Frondanol	Reduction of inflammation-associated changes in the colon in mice, reduction of cytokine content at the protein and mRNA level	[70]
Sea cucumber	Echinodermata	*Cucumaria frondosa*	Lipid	Sphingolipids	Inhibition of pro-inflammatory cytokines IL-1β, IL-6 TNF-α and increasing anti-inflammatory IL-10 via inhibition of phosphorylation of JNK and translocation of NF-κB	[71]
Sea cucumber	Echinodermata	*Cucumaria frondosa*	Lipid	Frondaol A5	Attenuation of circulating inflammatory cytokines and suppression of mRNA expression of inflammatory markers such as 5-LOX and FLAP	[72]
Sea urchins	Echinodermata	*Scaphechinus mirabilis*	Dark red pigment	EchA	Attenuation of macrophage activation and infiltration (neutrophils), inhibition of TNF-α and IFNγ in bleomycin-induced scleroderma mouse model	[73]
Sea urchins	Echinodermata	?	Dark red pigment	EchA	Decreasing DIA, improvement of colon length and suppression of tissue damage, suppression of macrophage activation	[74]
Sea urchins	Echinodermata	?	Dark red pigment	EchA	TNF-α and NF-κB inhibition in Lewis rats	[75]
Sea urchins	Echinodermata	*Paracentrotus lividus*	Dark red pigment	EchA	Potent stabilizing effect on the human red blood cells, suppression of the production of IL-6 and TNF-α in septic rats	[76]
Sea urchins	Echinodermata	*Scaphechinus mirabilis*	Pigment	Spinochrome A	Reduction of chronic inflammation in cotton-pellet granuloma rat model	[77]
Sea urchins	Echinodermata	*Scaphechinus mirabilis*	Pigment	Spinochrome B		[77]
Sea urchins	Echinodermata	*Echinometra mathaei*, *diadema savignyi*, *tripneustes gratilla* and *Toxopneustes pileolus*	Pigment	Spinochromes	TNF-α inhibition in J774 macrophages	[78]
Sea urchins	Echinodermata	*Echinometra mathaei*, *diadema savignyi*, *tripneustes gratilla* and *Toxopneustes pileolus*	Pigment	EchA		
Sea urchins	Echinodermata	*Strongylocentrotus droebachiensis*	Peptide	Centrocin 1 (CEN1HC-Br)	IL-12 p40, IL-6, IL-1β and TNF-α inhibition in THP-1 cells	[79,80]
Sea urchins	Echinodermata	*Salmacis bicolor*	Isochroman derived polyketide	Salmachroman	COX-2 and 5-LOX inhibition by using the 2, 7-dichlorofluorescein method	[81]
Sea urchins	Echinodermata	*Salmacis bicolor*	Polyoxygenated furanocembranoid derivatives	Salmacembrane A Salmacembrane B	COX-1, COX-2, and 5-LOX inhibition by the 2, 7-dichlorofluorescein method	[82]
Sea urchins	Echinodermata	*Stomopneustes variolaris*	Cembrane type of diterpene	4-hydroxy-1-(16methoxyprop-16-en-15-yl)-8-methyl-21,22-dioxatricyclo[11.3.1.15,8]octadecane-3,19-dione	Inhibition of 5-LOX, COX-1 and COX-2 inhibition by the 2, 7-dichlorofluorescein method	[83]
Sea urchins	Echinodermata	*Stomopneustes variolaris*	Macrocyclic lactone	Stomopneulactones D	COX-2, 5-LOX, iNOS inhibition in RAW 264.7 cells	[84]
Sea urchins	Echinodermata	*Brisaster latifrons*	Sulfonic acid	(Z)-4-methylundeca-1,9-diene-6-sulfonic acid	Inhibition of proinflammatory cytokines by the inactivation of JNK/p38 MAPK and NF-kB pathways	[85]
Sea urchins	Echinodermata	*Hemicentrotus pulcherrimus* and *Diadema setosum*	Lipid	Hp-s1 ganglioside	Inhibition of iNOS, COX-2, and cytokines, downregulation of the NF-κB and JNK/P38 MAPK signaling pathway	[86]
Ascidian	Chordata	*Aplidium orthium*	Alkaloids	Alkaloid tubastrine, Orthidine A, Orthidine B, Orthidine C, Orthidine E, Orthidine F	Reduction of the superoxide synthesis in PMA-stimulated neutrophils in vitro and in in vivo models	[87]
Ascidian	Chordata	*Aplidium* spp.	Alkaloids	Ascidiathiazone A, Ascidiathiazone B	Reduction of the superoxide production by PMA-stimulated neutrophils in vitro and in vivo in murine gout model	[88]
Ascidian	Chordata	*Pycnoclavella kottae*	Alkaloid	Kottamide D	Reduction of superoxide synthesis by PMA and N-formylmethionyl-leucyl-phenylalanine (fMLP)-activated neutrophils *in vitro*	[89]
Red algae	Rhodophyta	*Gracilaria opuntia*	Alkaloid	Azocinyl morpholinone	Inhibition of the carrageenan-induced paw edema	[90]
Green algae	Chlorophyta	*Enteromorpha prolifera*	Chlorophyll	Pheophytin	Suppression of the production of superoxide anion in mouse macrophages	[91]
Green algae	Chlorophyta	*Ulva lactuca*	Sterol	3-0-B-D-glucopyranosil-stigmata-5,25,-dien sterol	Topical anti-inflammatory activity in mouse edema	[92]
Green algae	Chlorophyta	*Caulerpa racemosa*	Alkaloid	Caulerpin//Sulfated polysaccharides	Inhibition of capsaicin-induced ear edema model and significant reduction of the number of recruited cells; reduction in neutrophil counts in the peritoneal cavity and paws of carrageenan-treated rats; reduction of edema volume in carrageenan and dextran-activated mouse paws	[93,94]
Green algae	Chlorophyta	*Enteromorpha prolifera*	Chlorophyll	Pheophytin A	Significant suppression of TPA-induced inflammatory reactions such as edema formation in BALB/c mouse ears	[91]
Green algae	Chlorophyta	*Caulerpa mexicana*	Sulfated polysaccharides	Sulfated polysaccharides	Reduction of edema volume and neutrophilic infiltration in carrageenan-activated raw paws; Reduction of edema volume in dextran and histamine-activated rat paws	[95]
Green algae	Chlorophyta	*Caulerpa cupressoids*	Protein	Lectin	Reduction of leukocyte counts and myeloperoxidase activity in rat temporomandibular joint synovial lavage fluid in zymosan-activated rats	[96]
Brown algae	Heterokontophyta	*Ecklonia cava*	Pholorotannin	Dieckol	Inhibition of NO, PGE2, and the expression of iNOS production in murine BV2 microglia	[97]
Brown algae	Heterokontophyta	*Undaria pinnatifida*	Fatty acid	Ω-3 polyunsaturated fatty acids	Inhibition of the mouse ear inflammation induced by PMA	[98]
Brown algae	Heterokontophyta	*Laminaria japonica*	Sulfated polysaccharide	Fucoidan	NO and IL-6 inhibition in Caco-2 cells	[99]
Brown algae	Heterokontophyta	*Fucus vesiculosus*	Sulfated polysaccharide	Fucoidan	Reduction of NO, PGE2, TNF-α and IL-1β production in RAW 264.7 cells	[100]
**Microorganisms**						
**Organisms**	**Classification** **(Phylum)**	**Species**	**Type of Molecules**	**Molecules**	**Target/Mode of Action**	**Ref(s)** **.**
Dinoflagellate (microalgae)	Dinoflagellata	*Symbiodinium* spp.	Amphoteric iminium	6,6,6-tricyclic iminium ring and aryl sulfate moiety	Inhibition of the COX-2 activity in RAW 264.7 cells	[101]
Haptophyte (microalgae)	Haptophyta	*Isochrysis galbana*	Galactolipids	Monogalactosyldiacylglycerols Digalactosyldiacylglycerol	Inhibition of the synthesis of TNF-α, IL-1β, IL-6, IL-17 in THP-1 cells	[102]
Green microalgae	Chlorophyta	*Chlorella vulgaris*	Polyunsaturated fatty acid	Linoleic acid and α-linolenic	Inhibition of TNF-α, IL-6, PGE2, and NO production in RAW 264.7 cells	[103]
Red microalgae	Rhodophyta	*Porphyridium cruentum*	Fatty acids	Fatty acids	Inhibition of superoxide anion production by peritoneal leukocytes primed with PMA	[104]
Red microalgae	Rhodophyta	*Porphyridium cruentum*	Exopolysaccharide (EPS)	EPS	Inhibition of 77% of COX-2 in human keratinocytes and murine fibroblasts Balb/c-3T3	[105]
			Pigment	Phycoerythrin	Inhibition of COX-2 in human keratinocytes and murine fibroblasts Balb/c-3T3	[105]
Cyanobacteria	Cyanobacteria	*Spirulina subsalsa*	Lipids (glycophospholipids, phospholipids)	Sulfoquinovosyl diacylglycerols, monogalactosylodiglycerides, cerebrosides; ceramides, phosphatidylcholines, phosphatidylethanolamines	Inhibitory effects on platelet-activating factor and thrombin-induced platelet aggregation	[106]
Cyanobacteria	Cyanobacteria	*Lyngbya majuscula*	Malyngamide	Malyngamide F acetate	Inhibition of the NO production in RAW 264.7 cells	[107]
Cyanobacteria	Cyanobacteria	*Caldora* sp.	Azirine	Dysidazirine carboxylic acid	Inhibition of the NO production by almost 50% at 50 µM in RAW 264.7 cells	[108]
Fungi	Ascomycota	*Chaetomium globosum QEN-14*	Alkaloid	Chaetoglobosin Fex	Inhibition of TNF-α and IL-6 production in LPS-activated RAW 264.7 cells	[109]
Fungi	Ascomycota	*Stachybotrys* sp. *HH1 ZSDS1F1-2* (isolated from a sponge from Xisha Island, China, in April 2012)	Xanthonne	Xanthone derivatives 3 (others),	Inhibition of COX-2	[110]
				Xanthone derivatives 4 (others),		
				Xanthone derivatives 11 (others)		
Fungi	Ascomycota	*Aspergillus* spp.	Diketopiperazine alkaloids	5-prenyl-dihydrovariecolorin F	Inhibition of iNOS and COX-2 activity, reduction of NO and PGE2 levels in LPS-stimulated RAW 264.7 and BV2 cells	[111]
Fungi	Ascomycota	*Aspergillus* spp.	Diketopiperazine alkaloids	5-prenyl-dihydrorubrumazine A		
Fungi	Ascomycota	*Aspergillus* sp. *SF-6354*	Polyketide	TMC-256C1	NO and PGE2 inhibition in LPS-activated BV2 cells	[112]
Fungi	Ascomycota	*Aspergillus* sp. *SCSIO Ind09F01*	Polyketides	Diorcinol, Cordyol C, 3,7-dihydroxy-1,9-dimethyldibenzofuran	Inhibition of COX-2 (IC_50_ = 2.4–10.6 µM)	[113]
Fungi	Ascomycota	*Aspergillus* sp. *SF-5974* and *Aspergillus* sp. *SF-5976*	Polyketides	Cladosporin 8-0-α-ribofuranoside, Cladosporin, Asperentin 6-O-methyl ether Cladosporin 8-O-methyl ether, 4′-hydroxyasperentin, 5′-hydroxyasperentin	Inhibition of NO and PGE2 expression, (IC_50_ = 20–65 µM) in LPS-activated microglial cells	[114]
Fungi	Ascomycota	*Aspergillus* sp. *SF-5044*	Polyketide	Asperlin	Inhibition of NO and PGE2 expression in LPS-activated murine macrophages	[115]
Fungi	Ascomycota	*Aspergillus* sp.	Peptide	Aurantiamide acetate	Inhibition of NO and PGE2 expression in LPS-activated BV2 cells	[116]
Fungi	Ascomycota	*A.* *europaeus WZXY-SX-4-1*	Polyketides	Eurobenzophenone B, Euroxanthone A, 3-de-O-methylsulochrin, Yicathin B, Dermolutein, Methylemodin	Inhibition of NF-κB activation and NO expression in LPS-activated SW480 cells	[117]
Fungi	Ascomycota	*Aspergillus* sp. *ZLO-1b14*	Terpenes	Aspertetranone A, Aspertetranone B, Aspertetranone C, Aspertetranone D	Inhibition of IL-6 expression in LPS-activated RAW 264.7 cells	[118]
Fungi	Ascomycota	*A.* *sydowii J05B-7F-4*	Polyketide	Violaceol II, Cordyol E	Inhibition of NO (IC_50_ = 73 µM) expression in LPS-activated RAW 264.7 cells	[119]
Fungi	Ascomycota	*A.* *niger SCSIO Jcsw6F30*	Polyketides	Aurasperone F, Aurasperone C, Asperpyrone A	Inhibition of COX-2 expression (IC_50_ = 11.1, 4.2, and 6.4 µM for F, C, and A, respectively) in LPS-activated RAW 264.7 cells	[120]
Fungi	Ascomycota	*A. flocculosus 16D-1*	Alkaloids	Preussin C, Preussin D, Preussin E, Preussin F, Preussin G, Preussin H, Preussin I, Preussin J, Preussin K	Inhibition of IL-6 expression in LPS-activated THP-1 cells	[121]
Fungi	Ascomycota	*A.* *versicolor*	Alkaloids	Asperversiamide B, Asperversiamide C, Asperversiamide F, Asperversiamide G	Inhibition of iNOS expression in LPS-activated RAW 264.7 cells	[122]
Fungi	Ascomycota	*A.* *terreus*	Alkaloid	Luteoride E	Inhibition of NO in LPS-activated RAW 264.7 cells	[123]
Fungi	Ascomycota	*A.* *terreus*	Terpene	Lovastatin	Inhibition of NO production in LPS-activated RAW 264.7 cells	[123]
Fungi	Ascomycota	*A.* *terreus CFCC 81836*	Terpene	Brasilanone A	Inhibition NO production in LPS-activated RAW 264.7 cells	[124]
Fungi	Ascomycota	*A.* *terreus CFCC 81836*	Terpene	Brasilanone E		[124]
Fungi	Ascomycota (phylum)	*A.* *terreus*	Polyketide	Versicolactone G	Inhibition of NO production (IC_50_ = 15.72 and 29.34 µM for G and A, respectively) in LPS-activated RAW 264.7 cells	[123]
Fungi	Ascomycota	*A.* *terreus*	Polyketide	Territrem A		
Fungi	Ascomycota	*A.* *terreus*	Peptide	Methyl 3,4,5-trimethoxy-2-(2-(nicotinamido)benzamido)benzoate	Inhibition of NO production in LPS-activated RAW 264.7 cells	[123]
Fungi	Ascomycota	*A. terreus* (isolated from the coral *Sarcophyton subviride*)	Aliphatic alcohol	(3E,7E)-4,8-dimethyl-undecane-3,7-diene-1,11-diol, 14α-hydroxyergosta-4,7,22-triene-3,6-dione	Inhibition of NO expression in LPS-activated RAW 264.7 cells	[123]
Fungi	Ascomycota	*Aspergillus* sp. *SCSIOW2*	Terpenes	Dihydrobipolaroxins B-D Dihydrobipolaroxin	NO inhibition in RAW 264.7 cells	[125]
Fungi	Ascomycota	*Eurotium* sp., *SF-5989*	Alkaloid	Neoechinulin B	Inhibition of NO production in amyloid-β 1-42-activated BV-2 cells	[126]
Fungi	Ascomycota	*Eurotium* sp. *SF-5989*	Polyketide	Flavoglaucin Isotecrahydroauroglaucin	Inhibition of NO and PGE2 expression in LPS-activated RAW 264.7 cells	[127]
Fungi	Ascomycota	*Eurotium* spp.	Indolic alkaloid	Neoechinulin A	Reduction of NO and PGE2 production by inhibiting iNOS and COX-2 expression and reduced the production of IL-1β, TNF-α in LPS-stimulated RAW 264.7 cells	[126]
Fungi	Ascomycota	*Eurotium* sp. *SF-5989*	Alkaloid	Neocechinulin A	Inhibition of NO and PGE2 in LPS-stimulated RAW 264.7 macrophages	[126]
Fungi	Ascomycota	*E.* *amstelodami*	Polyketide	Asperflavin	Inhibition of 4.6% and 55.9% of NO and PGE2 expression, respectively, in LPS-activated RAW 264.7 cells	[128]
Fungi	Ascomycota	*E.* *amstelodami*	Polyketide	Questinol	Inhibition of 73% and 43.5% of NO and PGE2 expression, respectively, in LPS-stimulated RAW 264.7 cells	[129]
Fungi	Ascomycota	*Penicillium* sp. *SF-5859* (isolated from a sponge)	Polyketides	Curvularin, (11R,15S)-11-hydroxycurvularin, (11S,15S)-11-hydroxycurvularin, (11R,15S)-11-methoxycurvularin, (11S,15S)-11-methoxycurvularin, (10E,15S)-10,11-dehydrocurvularin, (10Z,15S)-10,11-dehydrocurvularin	Inhibition of NO and PGE2 expression (IC_50_ values ranging from 1.9 to 18.7 µM) in LPS-stimulated RAW 264.7 cells	[130]
Fungi	Ascomycota	*Graphostroma* sp. *MCCC 3A00421*	Terpene	Graphostromane F	Inhibition of NO in LPS-activated RAW 264.7 cells	[131]
Fungi	Ascomycota	*Graphostroma* sp. *MCCC 3A00421*	Terpene	Khusinol B	Inhibition of NO expression in LPS-activated RAW 264.7 cells	[132]
Fungi	Ascomycota	*P.* *chrysogenum SCSIO41001*	Alkaloid	Chrysamide C	Inhibition of IL-17 expression in mice T-cells	[133]
Fungi	Ascomycota	*Penicillium* sp. *SF-5295*	Alkaloid	Viridicaol	Inhibition of NO and PGE2 expression in LPS-activated RAW 264.7 and in LPS-activated BV2 cells	[134]
Fungi Fungi	Ascomycota	*Penicillium* sp.	Alkaloids	Brevicompanine E, Brevicompanine H	Inhibition of NO production in LPS-activated RAW 264.7 cells	[135]
Fungi	Ascomycota	*Penicillium* sp. *SF-5995*	Alkaloid	Methylpenicinoline	Inhibition of NO, PGE2, iNOS and COX-2 expression in LPS-induced RAW 264.7 cells and BV2 microglia	[136]
Fungi	Ascomycota	*Penicillium* sp. *SF-5497*	Terpenes	7-acetoxydehydroaustinol, Austinolide, 7-acetoxydehydroaustin, 11-hydroxyisoaustinone, 11-acetoxyisoaustinone	Inhibition of NO expression in LPS-activated BV-2 cells	[137]
Fungi	Ascomycota	*Penicillium* sp. *SF 6013*	Terpenes	2E,4Z-tanzawaic acid D, Tanzawaicacids A, Tanzawaicacids E	Inhibition of NO expression in LPS-activated RAW 264.7 cells	[138]
Fungi	Ascomycota	*Penicillium* sp. *SF-5629*	Polyketide	Citrinin H1	Inhibition of NO and prostaglandin E2 expression (IC_50_ = 8.1 and 8.0 µM) in LPS-activated BV2 cells	[139]
Fungi	Ascomycota	*Penicillium* sp. *SF-5292*	Polyketide	Penicillospirone	Inhibition of NO and PGE2 expression (with IC_50_ values of 21.9–27.6 µM) in LPS-activated RAW 264.7 and BV2 cells	[134]
Fungi	Ascomycota	*Penicillium* sp. *SF-5292*	Polyketide	Penicillinolide A	Inhibition of NO, PGE2, TNF-α, IL-1β, and IL-6 expression (IC_50_ = 20.47, 17.54, 8.63, 11.32, and 20.92 µM, respectively) in LPS-activated RAW 264.7 and BV2 cells	[140]
Fungi	Ascomycota	*Penicillium* sp. *J05B-3-F-1*	Hexylitaconic acid derivatives	Methyl 8 -hydroxy-3-methoxycarbonyl-2-methylenenonanoate, (3S)-Methyl 9-hydroxy-3-methoxycarbonyl-2-methylenenonanoate	Inhibition of pro-inflammatory cytokines and NO expression in LPS-activated RAW 264.7 cells	[141]
Fungi	Ascomycota	*P. atrovenetum*	Terpene	Citreohybridonol	Anti-neuroinflammatory activity	[142]
Fungi	Ascomycota	*P.* *steckii 108YD142*	Terpenes	Tanzawaic acid Q, Tanzawaic acid A, Tanzawaic acid C, Tanzawaic acid D, Tanzawaic acid K	Inhibition of NO expression in LPS-activated RAW 264.7 cells	[143]
Fungi	Ascomycota	*P.* *paxililli*	Polyketide	Pyrenocine A	Inhibition of TNF-α and PGE2 expression in LPS-activated RAW 264.7 cells	[144]
Fungi	Ascomycota	*P.* *thomii KMM 4667*	Terpene	Thomimarine E	Inhibition of 22.5% of NO production in LPS-activated RAW 264.7 cells	[145]
Fungi	Ascomycota	*P.* *thomii KMM 4667*	Polyketide	Guaiadiol A, 4,10,11 trihydroxyguaiane	Inhibition of 24.1% and 36.6% of NO production at 10 µM in LPS-activated RAW 264.7 cells	[145]
Fungi	Ascomycota	*P.* *citrinum SYP-F-2720*	Peptide	(S)-2-(2-hydroxypropanamido)benzoic acid	Reduction of the inflammation in xylene-induced mouse ear edema model	[146]
Fungi	Ascomycota	*Hypocreales* sp. *HLS-104*	Terpene	1R,6R,7R,10S-10-hydroxy-4(5)-cadinen-3-one	Inhibition of NO expression in LPS-activated RAW 264.7 cells with Emax value of 26.46% at 1 µM	[147]
Fungi	Ascomycota	*Hypocreales* sp. *HLS-104*	Polyketide	(R)-5,6-dihydro-6-pentyl-2H-pyran-2-one		
Fungi	Ascomycota	*F.* *heterosporum CNC-477*	Sesterpene polyol	Mangicol A	Inhibition of PMA-induced mouse ear edema assay	[148]
Fungi	Ascomycota	*F.* *heterosporum CNC-477*	Sesterpene polyol	Mangicol B		
Fungi	Basidiomycota	*Chondrostereum* sp. *NTOU4196*	Sesquiterpenes	Chondroterpene A, Chondroterpene B, Chondroterpene H, Hirsutanol A, Chondrosterin A, Chondrosterin B	Inhibition of NO expression in LPS-activated BV-2 cells	[149]
Fungi	Ascomycota	*Pleosporales* sp.	Terpenes	Pleosporallin A, Pleosporallin B, Pleosporallin C	Inhibition of IL-6 expression in LPS-activated RAW 264.7 cells	[150]
Fungi	Ascomycota	*Phoma* sp. *NTOU4195*	Polyketide	Phomaketides A-C, FR-111142	Inhibition of NO expression (IC_50_ values ranging from 8.8 to 19.3 µM) in LPS-activated RAW 264.7 cells	[151]
Fungi	Ascomycota	*Stachybotrys chartarum 952*	Terpenes	Stachybotrysin C, Stachybonoid F, Stachybotylactone	Inhibition of NO expression in LPS-activated RAW 264.7 cells	[152]
Fungi	Ascomycota	*Leptosphaerulina chartarum 3608*	Polyketide	(4R,10S,4′S)-leptothalenone B	Inhibition of NO in LPS-activated RAW 264.7 cells (IC_50_ = 44.5 µM)	[153]
Fungi	Ascomycota	*Glimastix* sp. *ZSDS1-F11*	Polyketides	Expansol A, Expansol B, Expansol C, Expansol D, Expansol E, Expansol F	Inhibition of COX-1 (IC_50_ = 5.3, 16.2, 30.2, 41 and 56.8 µM, for A, B, C, E, F respectively) and COX-2 (IC_50_ = 3.1, 5.6, 3, 5.1, 3.2 and 3.7 µM, for A, B, C, D, E, F, respectively)	[154]
Fungi	Ascomycota	*Diaporthe* sp. *HLY-1*	Polyketide	Mycoepoxydiene	Inhibition of NO and TNF-α, IL-6, and IL-1β expression in LPS-activated macrophages	[155]
Fungi	Ascomycota	*Aspergillus violaceofuscus*	Peptides	Violaceotide A, diketopiperazine dimer	Inhibition of IL-10 expression in LPS-activated THP1 cells	[156]
Fungi	Ascomycota	*Acremonium* sp.	Peptide	Oxepinamide A	Inhibition of RTX-activated mouse ear edema assay	[157]
Fungi	Ascomycota	*Alternaria* sp.	Peptide	Alternaramide	Inhibition NO and PGE2 expression in LPS-activated RAW 264.7 and BV2 cells	[158]
Fungi	Ascomycota	*Trichoderma citrinoviride (isolated from green alga Cladophora)*	Sorbicillinoid	Trichodermanone C	Inhibitory effect on nitrite levels in LPS-activated J774A.1 macrophages	[159]
Fungi	Ascomycota	*Paraconiothyrium* sp. *VK-13*	Polyketide	1-(2,5-dihydroxyphenyl)-3-hydroxybutan-1-one, 1-(2,5-dihydroxyphenyl)-2-buten-1-one	Inhibition of NO and PGE2 expression in LPS-activated RAW 264.7 cells (IC_50_ = 3.9–12.5 µM).	[160]
Fungi	Basidiomycota	*Cystobasidium larynges IV17-028*	Phenazine derivatives	6-[1-(2-aminobenzoyloxy)ethyl]-1-phenazinecarboxylic acid, Saphenol, (R)-saphenic acid, Phenazine-1-carboxylic acid, 6-(1-hydroxyethyl)phenazine-1-carboxylic acid, 6-acetyl-phenazine-1-carboxylic acid	Inhibition of NO production in RAW 264.7 cells	[161]
Fungi	Ascomycota	*Penicillium sp JF-55 (polyketide)*	Phenylpropanoid	Penstyrylpyrone	Inhibition of NO, PGE2, TNF-α, IL-1β in LPS-activated murine peritoneal macrophages	[162]
Bacteria	Actinobacteria	*Streptomyces* spp.	Alkaloid	Actinoquinoline A	Inhibition of COX-1 and COX-2	[163]
				Actinoquinoline B		
Bacteria	Actinobacteria	*Streptomyces caniferus*	Macrolide	Caniferolide A	Inhibition of NF-κB p65 translocation and pro-inflammatory cytokines expression in BV2 microglial cells	[164]
Bacteria	Actinobacteria	*Nocardiopsis* sp.	Macrolide	Fijiolide A	Reduction of TNF-α-induced NF-κB in human embryonic kidney cells 293 (IC_50_ = 0.57 µM)	[165]
Bacteria	Actinobacteria	*Kocuria* sp. *strain AG5*	Exopolysaccharide	EPS5	Inhibition of LOX-5 and COX-2 (IC_50_ = 15.39 ± 0.82 µg/mL and 28.06 ± 1.1 µg/mL, respectively)	[166]
Bacteria	Bacillota	*Bacillus subtilis B5*	Macrolactin derivative	7,13-epoxyl-macrolactin A; 7-O-2′E-butenoyl macrolactin A	Inhibition of inducible nitric oxide synthase (iNOS), interleukin-1β (IL-1β), and interleukin-6 (IL-6) expression in LPS-stimulated RAW 264.7 macrophages	[167]

## 3. Marine Microorganisms vs. Macro-Organisms: Who Are the Actual Producers of Metabolites?

**Figure 1:**
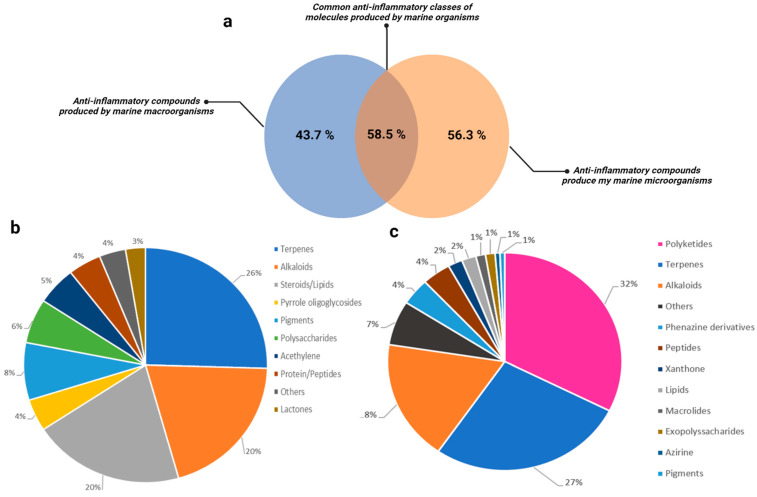
Chemical classification of MNPs with anti-inflammatory activity as reported between 2000 and 2024. Percentage of known anti-inflammatory compounds produced by marine organisms (**a**), by marine macro-organisms (**b**), and microorganisms (**c**) according to the structure type.

Oceans are a vast and unexplored world, teeming with life and diversity. Recent advancements in bioprospecting and molecular technologies foster the identification of new marine organisms, from macroscopic to microscopic biota, in this fascinating ecosystem [168]. However, the number of unknown marine species is estimated between 60,000 and 1,950,000, depending on the literature [169]. In the early days, bioprospecting campaigns focused on larger species like cnidarians, sponges, or soft corals due to technical limitations [170]. Between the 1990s and the 2010s, marine invertebrates have been found to produce almost 10,000 new marine natural products (MNPs) [171]. These discoveries have revealed the immense potential of marine organisms for developing innovative compounds for therapeutic and industrial applications. Many metabolites produced by marine macro-organisms have shown promising biological properties, such as anti-inflammatory activity for 43.7% of compounds (Figure 1a). These metabolites belong to different classes of molecules like terpenes (26%), alkaloids (20%), lipids (20%), pigments (8%), polysaccharides (6%) as shown in Figure 1b. Among macro-organisms, those belonging to the phylum Echinodermata produce the most anti-inflammatory molecules (Table 1), inhibiting pro-inflammatory cytokines and the NF-κB pathway but also reducing inflammation in vivo (Table 1). Since then, the possibility of further exploring and leveraging marine ecosystems has been genuinely exciting as it could unlock countless benefits for human health.

An ongoing exploration of marine ecosystems has extended to extreme environments such as deep ocean trenches, geographical poles, or hydrothermal vents; furthermore, technological improvement of microorganisms conservation during collects prompted bioprospecting campaigns to focus on microorganisms such as microalgae, marine fungi, cyanobacteria, and other groups of marine microorganisms. These microscopic life forms represent over 90% of the marine biomass and play a critical role in geochemical processes necessary for terrestrial life [172]. They are also remarkable for their ability to thrive, even in the harshest environments, producing rare and unique compounds that cannot be found in terrestrial biotopes. Furthermore, marine microorganisms are highly metabolically efficient, producing large amounts of metabolites while consuming limited energy [173]. Over the past year, MNPs obtained from marine bacteria, fungi, and cyanobacteria increased by 22%, 85%, and 61%, respectively, between 2018 and 2020, underscoring the impact of marine microorganisms on scientific research [174]. Yet, macro-organisms such as sponges and cnidarians have also been shown to produce MNPs [175]. The identification of these sources has led to inquiries and discussions about the actual producers of these metabolites.

Recent studies have uncovered that certain compounds previously thought to be specifically produced by marine macro-organisms are actually the metabolic byproducts of associated microorganisms [176], as illustrated by bryostatin, which has been confirmed to originate from microbes. The discovery of this metabolite has been made through the identification of polyketide synthase genes involved in its biosynthesis and found in the genome of the bryozoan bacterial symbiont *Candidatus Endobugula sertula* [177]. Another striking example is the fungus *Penicilium canescens* found in the ascidian *Styela plicata*, which exhibited anti-inflammatory activity. Furthermore, the findings presented in Figure 1a indicate that 58.3% of common anti-inflammatory classes of molecules are produced by both marine macro-organisms and microorganisms. This suggests that microorganisms may play a crucial role in producing these compounds, as many microorganisms live in symbiosis with macro-organisms.

**Figure 2:**
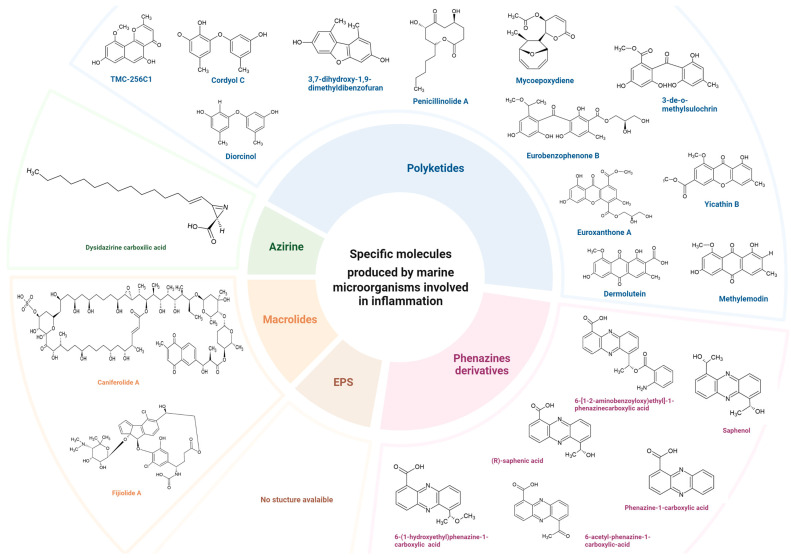
Chemical structure of specific molecules produced by marine microorganisms according to their classification. Regarding polyketides, only a few molecules were presented for each specific target involved in inflammation.

In comparison with macro-organisms, microorganisms represent a significant source of anti-inflammatory molecules, contributing a noteworthy 56% of these compounds (Figure 1a). Moreover, the diversity of their metabolites is astounding, including terpenes (27%), alkaloids (18%), peptides (4%), lipids (2%), and pigments (1%) as indicated in Figure 1C. However, the most intriguing aspect is the specific type of molecules, such as polyketides (32%) and phenazine derivatives (4%) produced by marine fungi that target pro-inflammatory cytokines like TNF-α or IL-6, as well as inflammatory markers like NO (Table 1, Figure 2). Given that these mediators are produced upon activation of the NF-kB pathway or are involved in the activation of the JAK-STAT pathway, it is plausible that the MNPs derived from fungi may inhibit these pathways. Additionally, marine microorganisms, particularly bacteria, can produce specific compounds that are not found in macro-organisms. These compounds, such as exopolysaccharides, macrolides, and azirine, can target inflammatory mediators such as cyclooxygenases, NO, TNF-α, and the NF-κB pathway (Table 1, Figure 2). It is worth noting that among microorganisms, most of the compounds are produced by fungi, particularly those belonging to the Ascomycota phylum (Table 1). In addition, they are the major producers of polyketides, one of the specific molecules mentioned above. Furthermore, although most specific molecules targeted the NF-κB pathway (Table 1), their structural characteristics prompt consideration of whether their modes of action could reveal new pathways and targets for modulating inflammation, thus extending our understanding of the interplay between marine compounds and the inflammatory process. These results suggest that fungi could potentially serve as valuable sources of anti-inflammatory molecules.

Considering the vast potential of microorganisms in the production of anti-inflammatory compounds, further research must be conducted to unlock their full potential and develop new treatments for inflammatory diseases.

## 4. Challenges and Future Directions

Exploring the potential of marine microorganisms as anti-inflammatory agents presents a myriad of challenges and promising future opportunities. One significant challenge lies in the development of anti-inflammatory drugs derived from marine sources, which may encounter barriers impacting the speed and efficiency of the process. Additionally, regulatory hurdles could potentially impede the approval and commercialization of marine-derived pharmaceuticals for anti-inflammatory purposes. Scaling up the production of bioactive compounds from marine microorganisms to meet demand poses a significant challenge, while ensuring the cost-effectiveness of extracting and utilizing these compounds for anti-inflammatory therapies is a critical consideration. The intricate complexity of marine ecosystems and the vast diversity of microorganisms further address the challenges in identifying and isolating effective anti-inflammatory compounds.

Looking towards the future, the quest for potent and effective anti-inflammatory natural products from marine organisms requires ongoing and rigorous research. It is essential to explore innovative approaches in marine drug discovery to uncover new and promising anti-inflammatory compounds. In the future, efforts should be focused on optimizing the drug development process from marine sources to enhance its efficacy and speed. Collaboration among researchers, industry members, and regulatory bodies is crucial for advancing marine-based anti-inflammatory therapies. Furthermore, emphasizing sustainable harvesting practices for marine microorganisms intended for anti-inflammatory purposes is vital for ensuring long-term viability.

By addressing these challenges and focusing on future directions, we can unlock marine microorganisms’ full potential as valuable sources of anti-inflammatory agents, leading to significant advancements in healthcare and therapeutic treatments.

## 5. Conclusions

**Figure 3:**
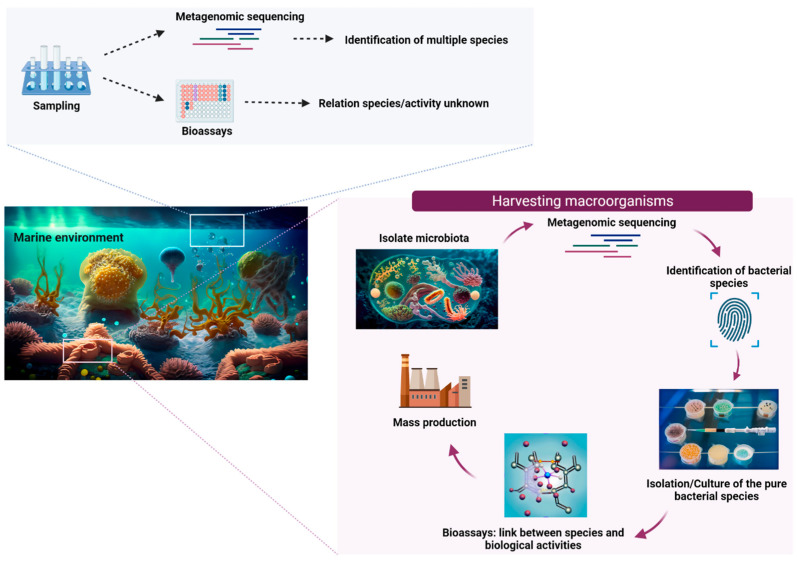
Metagenomic approach to discover the metabolites produced by the microbiota of marine macro-organisms. Two strategies are illustrated. In the top figure, whole metagenomics sequencing enables the identification of most species present in a microenvironment without driving the determination of a species/activity relationship. In the bottom part, microbiota isolation from the environment or macro-organisms leads to bacterial identification, specific culture, and a possible link between a metabolite and bioactivity.

The inter-relations between microorganisms and macro-organisms are complex, ranging from parasitic to symbiotic systems. In this regard, metagenomic analysis offers major insights to decipher the complexity of a micro-environment comprising a macro-organism and its hosts without providing any clues as to which among the various interacting, living species is actually responsible for the synthesis of the bioactive metabolites (Figure 3). On the other hand, microbiota identification and microbial isolation from a macro-organism is an attractive alternative, enabling the isolation and identification of specific bacterial species, their culture, and, ultimately, the demonstration of their ability to produce compounds of pharmaceutical interest. Indeed, microorganisms have emerged as a promising avenue for drug discovery, offering a solution to the challenges posed by low quantities of secondary metabolites and the difficulty of obtaining sufficient biomass necessary for pharmaceutical companies to perform clinical trials. Bacterial or microalgal cultures can provide a continuous source of biomass production within a subsequent purification of bioactive metabolites. These steps could revolutionize drug discovery by making it also more environmentally friendly by reducing the exploitation of marine resources.

## Data Availability

All data in this review are openly available without any restrictions.
